# Expression of sucrose metabolizing enzymes in different sugarcane varieties under progressive heat stress

**DOI:** 10.3389/fpls.2023.1269521

**Published:** 2023-10-16

**Authors:** Faisal Mehdi, Xinlong Liu, Zunaira Riaz, Urooj Javed, Afsheen Aman, Saddia Galani

**Affiliations:** ^1^ Sugarcane Research Institute, Yunnan Key Laboratory of Sugarcane Genetic Improvement, Yunnan Academy of Agricultural Sciences, Kaiyuan, China; ^2^ Agriculture and Agribusiness Management, University of Karachi, Karachi, Pakistan; ^3^ Dr. A. Q. Khan Institute of Biotechnology and Genetic Engineering (KIBGE), University of Karachi, Karachi, Pakistan; ^4^ National Key Laboratory for Biological Breeding of Tropical Crops, Yunnan Academy of Agricultural Sciences, Kunming, China; ^5^ Dow College of Biotechnology, Dow University of Health Sciences, Karachi, Pakistan

**Keywords:** heat stress, sugarcane, sucrose content, thermotolerance potential, sucrose metabolizing enzymes, stress damage indicators

## Abstract

Studying the thermal stress effect on sucrose-metabolizing enzymes in sugarcane is of great importance for understanding acclimation to thermal stress. In this study, two varieties, S2003-US-633 and SPF-238, were grown at three different temperatures ( ± 2°C): 30°C as a control, 45°C for various episodes of high temperature treatments and recovery conditions at 24, 48 and 72 hours. Data showed that reducing sugar content increased until the grand growth stage but sharply declined at the maturity stage in both cultivars. On the other hand, sucrose is enhanced only at the maturity stage. The expression of all invertase isozymes declined prominently; however, the expression of SPS was high at the maturity stage. Hence, the sucrose accumulation in mature cane was due to increased SPS activity while decreased invertase isozymes (vacuolar, cytoplasmic and cell wall) activities at maturity stage in both cultivars. Heat shock decreased the sucrose metabolizing enzymes, sucrose content and sugar recovery rate in both cultivars. In contrast, heat-shock treatments induced maximum proline, MDA, H_2_O_2_ and EC in both cultivars. Notably, this is the first report of diverse invertase isozyme molecular weight proteins, such as those with 67, 134 and 160 kDa, produced under heat stress, suggesting that these enzymes have varied activities at different developmental stages. Overall, S2003-US-633 performs better than the cultivar SPF-238 under heat stress conditions at all development stages, with increased sucrose content, enzyme expression, proline and sugar recovery rate. This work will provide a new avenue regarding sugarcane molecular breeding programs with respect to thermal stress.

## Introduction

Sugarcane is an important cash crop in Pakistan as well as in many countries, such as Brazil (37%), followed by India (18.7%), China (10.8%), Thailand (5.2%) and Pakistan (3.3%) (FAO, 2021). However, high temperatures due to climate change pose a significant threat to agricultural crop growth and development (Priya et al., 2019; Venkatesh et al., 2022). This fluctuating climate can impair plant growth and limiting the crop yield (2.5–10%) of major crops (Hatfield et al., 2011; Hassanein et al., 2012). The Intergovernmental Panel on Climate Change (IPCC) estimated that over the past two decades, the world’s temperature has risen by 1.09°C (IPCC, 2023). Weather and climate-related events are key factors in the production of sugarcane (Zhao et al, 2015). A rise in temperature of even 1°C beyond the optemum level is considered heat shock (Hasanuzzaman et al., 2013). This unfavorable temperature may affect the photosynthesis machinery, respiration and cell membrane thermostability (Kaushal et al., 2016). Proline, hydrogen peroxide, MDA and cell membrane thermostability (Demidchik et al., 2014) are the stress damage indicators under different environmental conditions. The net sucrose content in sugarcane stalks depends on the equilibrium between sucrose synthesis by sucrose synthysis enzyme groups such as sucrose synthase (SS) and sucrose phosphate synthase (SPS) and sucrose cleavage enzyme groups called invertase isoenzymes, including cell wall invertase (CWIN), cytoplasmic invertase (CyIN) and vacuolar invertase (VIN). SPS synthesizes fructose 6-phosphate, which is then dephosphorylated into sucrose by sucrose phosphate phosphatase (Hubbard et al., 1989; Barbier et al., 2019). Sucrose synthase enzymes either hydrolyze or synthesize sucrose in sugarcane plant leaves. The expression of SPS, SS and invertase isozymes declined when temperatures increased to 42°C (Gomathi et al., 2021). Conversely, in thermotolerant cultivars, SPS and SS expression levels were higher (Kohila and Gomathi, 2018). When temperatures increase, plants produce reactive oxygen species (ROS), which are extremely reactive and unstable, leading to oxidative stress and cell death, resulting in a reduction in yield and sucrose content (Waszczak et al., 2018). Oxidative stress can cause increased lipid peroxidation and protein and enzyme denaturation (Meriga et al., 2004). The role of proline is to maintain the water content in plant cells under heat stress (Singh et al., 2015).

Sugarcane crop improvement against environmental stress in the future will be essential for yield stability; an understanding of the biochemical and biological role of sucrose metabolizing enzymes and their interaction with heat stress is required. In recent years, sugarcane has been subjected to demanding research focused on enhancing its flexibility against heat stress; especially, the thermotolerant mechanisms and role of sucrose metabolizing enzymes under heat stress have become fundamental areas for enhancing sucrose content in sugarcane. Sugarcane has four growth stages: (i) formative or vegetative, (ii) tillering, (iii) grand growth and (iv) ripening or maturity stage (Gascho, 1985). In Pakistan, during the hot season (May–August), sugarcane is 150 to 250 days of age and in the grand growth stage, which is significant for actual cane formation, elongation and yield increase. Therefore, high temperatures at this time may affect growth, yield, sucrose metabolizing enzymes and sucrose accumulation in sugarcane stems during the maturity stage. Abiotic stresses at the grand growth stage cause a variable drop in cane and sugar production but a continual decline in sucrose content (Wiedenfeld, 2000). During the grand growth stage, tiller formation and development occur, together with shoot length and basal sucrose accumulation and thus this is known to be a critical stage for heat sensitivity due to the plant demanding the optimum temperature for growth and maximum sucrose concentration (Zingaretti et al., 2013).

The frequency and severity of extreme climatic circumstances are likely to rise owing to climate change, which might have had a detrimental impact on sugarcane output and will likely continue to do so. The intensity of the heat stress impact on sugcrane is interconnected with location and thermotolerance. But Kumudini et al. (2014) discovered that there hasn’t been much research to support these effects. Another study also reported that some cultivars were found to be thermotolerant under harsh environmental conditions (Zhao et al., 2015). According to our understanding, compared to the other abiotic factors, sugarcane heat stress has received significantly less study. The present study’s goal was to investigate the effect of heat stress on sucrose-metabolizing enzymes in sugarcane at different phenological phases. In addition, stress damage indicators such as lipoperoxidation,proline, hydrogen peroxide (H_2_O_2_) and cell membrane thermostability were analyzed to gain a better understanding of their thermotolerance potential. This work can contribute to screening out high-yielding, maximum sucrose accumulation, thermotolerant sugarcane and climate-resilient sugarcane variety development by understanding heat stress mechanisms and the role of sucrose metabolizing enzymes.

## Materials and methods

### Study plan

A pot experiment was conducted on two different local varieties of sugarcane, S2003-US-633 (high sucrose accumulation) and SPF-238 (low sucrose accumulation), at the KIBGE University of Karachi, Karachi, Pakistan, during the years 2016–2019. Sugarcane plants were grown in pots with 25 kg of loamy soil and 5 kg of fertilizer (farmyard manure). The treatment groups were divided into the following sequences by orders control plants were given at a temperature of 30°C ( ± 2°C), heat shock treatments were given at a temperature of 45°C ( ± 2°C), (24, 48 and 72 hour) and recovery treatments were given at a temperature of 30°C ( ± 2°C) (24, 48 and 72 hours). These treatments were given over periods of 24, 48 and 72 hours. Plants were subjected to heat stress in a heat shock room where photosynthesis-active radiation was maintained, ranging from 650–700 µmol m^-2^ s^-1^ during day and night (16 hours of light and 8 hours of darkness). The plants were shifted from control conditions into the heat shock room for heat shock treatments. Whereas for recovery treatments, plants shifted from heat shock rooms to field conditions for the above-mentioned period at vegetative, grand growth and maturity stages (during each treatment, samples were taken at 150, 250 and 350 days). All agronomic procedures were carried out during the trial. During heat shock treatments, constant water was applied to avoid drought and maintain humidity by installing a humidifier in the heat shock room. A totally random block design with three replications was used to set up the experiment.

### Sample collection

Leaf tissue was collected under controlled heat shock and recovery treatments at all phenological stages [vegetative (150 days), grand growth (250 days) and maturity (350 days)], then stored at −70 °C for further analysis.

### Morphological analysis

The shoot and root length were measured in terms of centimeters (cm). For the fresh-to-dry weight ratio, the plant’s fresh weight was measured on the day of sampling by using a weigh balance, placed in the oven for a week at 60°C and then measured for dry weight. The fresh-to-dry weight ratio was calculated by the following formula:


% of Moisture Loss = Fresh weight - Dry weight/Dry weight × 100

### Stress damage indicators analysis

#### Malondialdehyde (MDA) content determination

A membrane lipid peroxidation byproduct known as malondialdehyde (MDA) was measured to determine its level. For this, 0.1g of leaf tissue was extracted with tetracholoroacetic acid (1.5 ml). Centrifuged at 12,000 rpm for 10 minutes. A reaction mixture consisting of a sample (1 ml) and thiobarbituric acid (1 ml) was submerged for 30 minutes at 95°C in hot water. Then transfer to an ice bath. Again, after centrifuging, the optical density was measured at 532 and 600nm (Heath and Packer, 1968).

##### ✔ Calculation

Lipid peroxidation was expressed as µmolg^-1^ FW using the formula;


$$\text{MDA }=\frac{\text{A}532\;-\text{ A}600}{15500\;\times \;10^{6}}$$

#### Electrolyte leakage (EC)

Electrolyte leakage (EC) is used to determine relative membrane permeability (RMP), which is expressed as a percentage (%). For EC assessment, cut 0.5g of leaf tissue into 20 ml of water after being vortexed and placed at room temperature for 1 minute. Initial electrical conductivity (ECo) was calculated on the same day of sampling. Then all the tubes were placed in a 4°C refrigerator overnight. Then the next day, electrical conductivity (EC1) was calculated and lastly, EC2 was measured after autoclaving (Yang et al, 1996).

#### Hydrogen peroxide (H_2_O_2_)

For this 100 mg of leaf tissue was homogenized with 0.1% trichloroacetic acid (2.5 ml). Centrifuged at 12,000 rpm for 10 minutes. Sample (1 ml), phosphate buffer (1 ml) and potassium iodide (1.5 ml) were used in the reaction solutions. After being left at room temperature for ten seconds, the optical density of 390 nm was determined (Loreto and Velikova, 2001).

#### Proline

Sugarcane proline (Osmolytes accumulation) was measured using the method developed by Bate and his colleagues (Bates et al, 1973). A homogenate of leaves (0.1 g) and sulphosalicylic acid (2 ml) was used to estimate the free proline content. The sample was centrifuged at 10,000 rpm for 15 minutes. The supernatant, ninhydrin reagent and acetic acid were all added to the 3 ml reaction mixture in the same amount. Heated at a hundred degrees for 60 minutes, then kept on ice to stop the reaction. After the cooldown, toluene (5 ml) was added. After being vortexed and placed at room temperature for half an hour, the upper layer of the reaction mixture was collected and the absorbance was measured at 520nm.

#### Total soluble protein quantification

Total soluble proteins were calculated through the Bradford Assay (Bradford, 1976). The 3 ml reaction mixture containing 50 µl protein sample, Bradford dye 150 µl and 0.15 N NaCl 2800 µl was vortexed for 5 to 10 seconds, then incubated at room temperature for 15-20 minutes and read at 595 nm by spectrophotometer. A protein standard curve was constructed using a known concentration of BSA (10 to 100 µg ml^-1^).

### Sugar analysis

Total sugar analysis was assessed using the Anthrone method (Hedge et al, 1962). Briefly, 0.01g of sugarcane stem tissues were homogenized in 80 percent ethanol. For ten minutes, the extraction was centrifuged at 1000 rpm. Anthrone reagent was mixed. On a UV-1600 spectrophotometer (Tomos Life Science Group, China), the amount of total sugar was determined at 620 nm. For reducing sugar, 3,5-dinitrosalicylic acid (DNSA) was used. The optical density was measured using a spectrophotometer at 546nm (Miller, 1959). Nonreducing sugar was estimated using the following formula:


$$\text{Non-reducing sugar }\left({\text{mg g}}^{-1}\text{FW}\right)\text{ = total sugar-reducing sugar}$$

### Extraction of sucrose metabolizing enzymes

Frozen stem tissues (10g) were powdered in a mortar with liquid nitrogen, followed by continuous homogenizing in a 10 ml MOPS-NaOH buffer (pH 7.0). After centrifugation at 12000 rpm for half an hour, the supernatant was concentrated using a centricon centrifugal device. Enzymetic activity was quantified using the supernatant.

### Sucrose phosphate synthase and sucrose synthase analysis

Enzyme activity was quantified according to Huber et al. (1989), with some modifications. Briefly, for SPS activity, the reaction mixture (35 μl) containing 50 mM MOPS-NaOH (pH 7.5) buffer, 5 mM MgCl_2_, 1 mM EDTA, 2 mM uridine diphosphate glucose (UDPG), 4 mM fructose 6-P, 20 mM glucose 6-P and 10 μl of supernatant was incubated at 37°C for 20 min. The reaction was terminated using 70 µl of KOH (30%) and heated for 10 min at 95°C. Finally, 5 ml of anthrone reagent was added and incubated at 100°C for 20 min. The absorbance was measured at 620 nm. The protocol followed for the SS was similar to the SPS, except 10 mM fructose was used in the reaction mixture as a substrate instead of fructose 6-P and glucose 6-P.

### Invertase isozymes analysis

#### Quantitative analysis

To determine cell wall invertase activity, the reaction mixture contained 500 µl plant extract, 250 µl deionized water and 250 µl 4% sucrose in 0.05 M potassium acetate buffer (pH-3.5). The reaction mixture was incubated at 37°C for 60 minutes and neutralized by adding the 3, 5-dinitro salicylic acid (DNS) method. The above enzyme assay was similar to that for cytoplasmic invertase and vacoular invertase, except 0.1 M potassium-phosphate buffer (pH-7.0) for cytoplasmic invertase (Hatch et al., 1963) and 0.1 M potassium-phosphate buffer (pH-5.0) for vacuolar invertase (Vorster and Botha, 1999) were used in the assay.

Sucrose-metabolizing enzyme activities were calculated using the formula:


$$\text{Specific activity }=\frac{\text{ODT}\times \text{Conc Std }\left(\text{mg}\right)}{\text{OD Std }\times \text{AS}\times \text{MW}\times \text{RT}\times \text{TSP }}$$

Note: The unit of invertase isozymes (CWIN,CyIN and VIN) specific activity is (nmol hexose min^-1^ mg^-1^ protein), while for the SPS and SS (nmol sucrose min^-1^ mg^-1^ protein).

#### Zymographical analysis

Extracted isozymes were resolved on a NATIVE-PAGE gel (12%). After resolving, the gel was washed with deionized water, followed by enzyme activation in different buffers with their respective isozymes. Briefly, potassium phosphate buffer (pH 5.0) was used for vacuolar invertase, potassium phosphate buffer (pH 3.5) for cell wall invertase and potassium phosphate buffer (pH 7.0) for cytoplasmic invertase. The gel was incubated with 20% sucrose at 37°C overnight with slight shaking. The gel was washed with buffer and hexose sugar was visualized by soaking in a (1%) 2,3,5-triphenyl tetrazolium chloride (TTC) and NaOH (4%) solution (Arndt et al, 2012).

#### Sugar recovery rate estimation

The sugar recovery rate was estimated according to Foster et al. (1980). A crusher machine was used to quickly smash 1 kilogram of sugarcane stalks. The material was split into two pieces: 400g and 50g. The 400 g of crushed samples were mixed with 4 liters of water and then dissolved for half an hour. Lead (Pb) powder (1g) was added to the extraction (150 ml) and filtered. A 50-gram crushed sample was kept in an oven for 60 minutes at 150 °C.Then pol, °Brix, moisture content, fiber contents and recovery rate were measured by the following formulae:


Pol cane (%) = (extraction pol×0.26 (water+cane−(0.0125fiber×cane) / (specific gravity ×cane)

°Brix cane =(extraction brix×water−(0.25cane)−(0.0125cane×moister)/(cane×1−(0.0125×extraction brix)

Moister Content =Fresh Weight-Dry Weight-Tare Weight

Fiber Content (%) = 100−moisture-calculated °brix

Sugar recovery rate (%) =Pol −2.5

Note: (2.5% is the operating loss in sugar mills during processing, which varies depending on the mill).

### Statistical analysis

Data statistical analysis was done with a two-way ANOVA using R software, version 4.2.2. Multiple comparison analysis was performed by the Duncan test, and significance was assumed at *p<0.05.* Correlation analysis was done using SPSS software (version 23). Bar graphs were made using Excel.

## Results

### Stress damage indicators analysis

#### Electrolyte leakage (EC)

**Figure 1:**
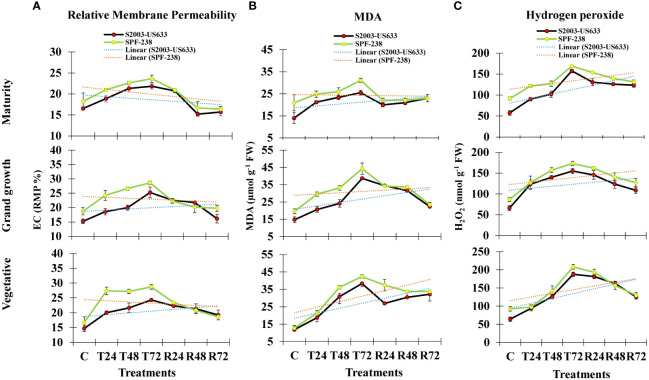
The bar plot represents thermotolerance indicator analysis **(A)** relative membrane permeability **(B)** MDA and **(C)** hydrogen peroxide (H_2_O_2_) of sugarcane cultivars S2003-US-633 and SPF-238 under control (C) at 30 ± 2°C, heat shock at 45 ± 2°C (T24, T48 and T72) and recovery at 30 ± 2°C (R24, R48 and R72) for 24, 48 and 72 hours at various phenologies. The values are the means and standard errors of three replicates. For each growth stage, the bars denote a significant difference with *p< 0.05*.

Electrolyte leakage (EC) is used to determine relative membrane permeability (RMP), which is expressed as a percentage (%). Electrolyte leakage is a stress indicator related to cell membrane injury and thermostability under heat stress. In untreated plants, the amount of EC was 14% in variety (S2003-US-633) and 16% in variety (SPF-238), but after heat stress exposure, EC content increased by 20% and 27% in both cultivars, respectively. The same result was observed at the grand growth stage. However, at maturity, EC content gradually declined in both cultivars. Comparatively, the maximum EC content was observed in cultivar SPF-238, which showed susceptible behavior under heat stress conditions (**Figure 1A**).

#### Malondialdehyde (MDA)

Lipidperoxidation is a extensively used thermotolerant indicatos in plant cell (Taulavuori et al., 2001). In the current study, MDA is produced as a result of lipid peroxidation and its content showed significant differences in both cultivars at all growth stages. At the vegetative stage, under heat stress conditions, the MDA content in cultivar SPF-238 steadily increased after 24 hours, 48 hours and 72 hours. Cultivar SPF-238 found higher MDA during heat stress conditions compared to S2003-US-633. Both cultivars displayed a comparable pattern of MDA buildup at the grand growth and maturity phases after recovery. Comparatively, cultivar S2003-US-633 showed the lowest MDA content as well as more rapid and better recovery than SPF-238 (**Figure 1B**).

#### Hydrogen peroxide (H_2_O_2_)

After exposure to heat stress, its content rapidly increased with increasing temperatures. In SPF-238, hydrogen peroxide accumulation was higher than in another cultivar. The same results were shown at the grand growth and maturity stages, but the vegetative stage was severely affected by heat stress in both cultivars. Variety S2003-US-633 had a quick recovery in the recovery state, which indicated that cultivar S2003-US-633 was showing tolerance in the heat shock condition (**Figure 1**).

#### Proline estimation

**Figure 2:**
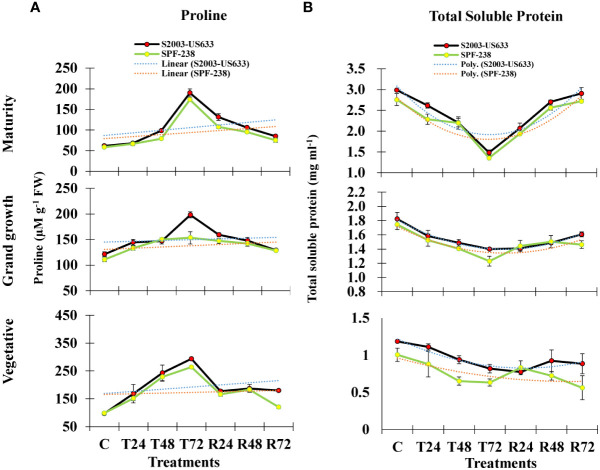
The bar plot represents thermotolerance indicator analysis **(A)** proline **(B)** total soluble protein of sugarcane cultivars S2003-US-633 and SPF-238 under control (C) at 30 ± 2°C, heat shock at 45 ± 2°C (T24, T48 and T72) and recovery at 30 ± 2°C (R24, R48 and R72) for 24, 48 and 72 hours at various phenologies. The values are the means and standard errors of three replicates. For each growth stage, the bars denote a significant difference with *p< 0.05*.

In the present study, exposure to heat stress led to a noticeably higher proline accumulation than the control level. It is noteworthy to notice that stress-tolerant sugarcane cultivars of S2003-US-633 have greater (293 µM g^-1^ FW) proline accumulation than SPF-238 (264 µM g^-1^ FW) over the untreated control plants, S2003-US-633 and SPF-238 had the lowest proline accumulation at the heat-stressed maturity stage (186 µM g^-1^ FW and 176 µM g^-1^ FW, respectively) and the pattern was seen at the grand growth stage as well. The thermotolerance index of proline accumulation between both sugarcane cultivars was studied. Under heat stress, cultivar S2003-US-633 accumulated proline considerably more than variety SPF-238 (**Figure 2**).

#### Total soluble protein quantification

Total soluble protein is also severely affected by heat stress conditions in both cultivars. However, S2003-US-633 had better or quicker response as compared to SPF-238 (**Figure 2B**).

### Quantitative analysis of sucrose metabolizing enzymes

#### Sucrose synthase (SS) activity

**Figure 3:**
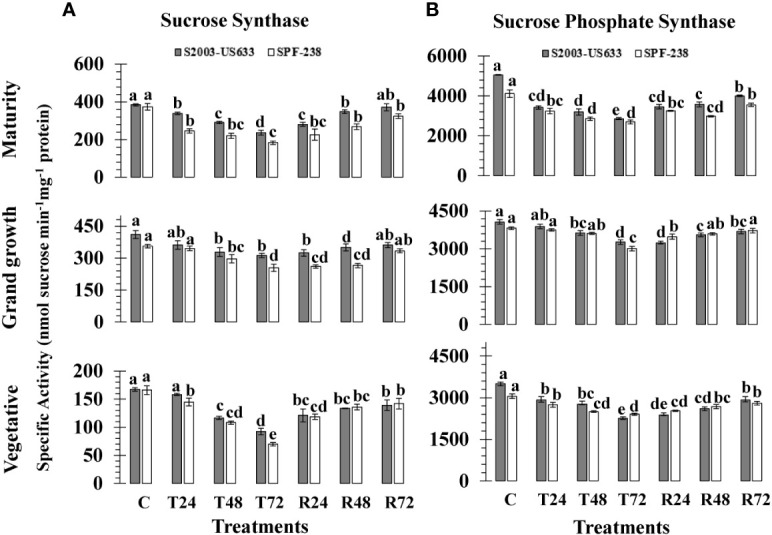
The activity of the enzymes **(A)** sucrose synthase and **(B)** sucrose phosphate synthase was measured in sugarcane cultivars S2003-US-633 and SPF-238 under the following conditions: Control (C) at 30 ± 2°C, heat shock at 45 ± 2°C (T24, T48 and T72) and recovery at 30 ± 2°C (R24, R48 and R72) for 24, 48 and 72 hours at various phenologies The values are the means and standard errors of three replicates. For each growth stage, a different letter above the bars denotes a significant difference with *p< 0.05*.

At different phenologies, the SS activity of sugarcane cultivars was assessed; the results are shown in **Figure 3A**. There was inconsistency in the SS activity in both varieties during heat shock treatments. The resistant S2003-US-633 showed the maximum SS activity under high-temperature stress circumstances (167.36 nmol sucrose min^-1^mg^-1^ protein), whereas SPF-238 recorded the lowest SS activity (166.16 nmol sucrose min^-1^mg^-1^ protein). The mean SS activity, % decline over the control, was lower in thermotolerant varieties S2003-US-633 (25%), and it declined % in susceptible varieties SPF-238 (35%). From the vegetative to the mature stages, SS activity increased.

#### Sucrose phosphate synthase (SPS) activity

Heat shock exposure had a detrimental impact on sucrose phosphate synthase and the specific activity of enzyme values in terms of (nmol sucrose min^-1^ mg^-1^ protein), of both sugarcane varieties at all phenological stages are presented in (**Figure 3B**). At control conditions, sugarcane varieties S2003-US-633 (3509.31 nmol sucrose min^-1^mg^-1^ protein) and SPF-238 (3054.75 nmol sucrose min^-1^mg^-1^ protein) had the highest SPS activity at the vegetative stage. When the sugarcane plants were exposed to high temperature stress at 45°C (± 2°C), a substantial decline in SPS-specific activity was exhibited in both varieties. The same results were also exhibited at the grand growth stage, which is higher than the vegetative stage. At the maturity stage, greater activity of SPS was observed in the thermotolerant variety S2003-US-633 (2858.04 nmol sucrose min^-1^mg^-1^ protein) than at the other two stages. At all growth stages, it was shown that the thermotolerant S2003-US-633 had more SPS activity than the susceptible SPF-238.

#### Cell wall invertase (CWIN) activity

**Figure 4:**
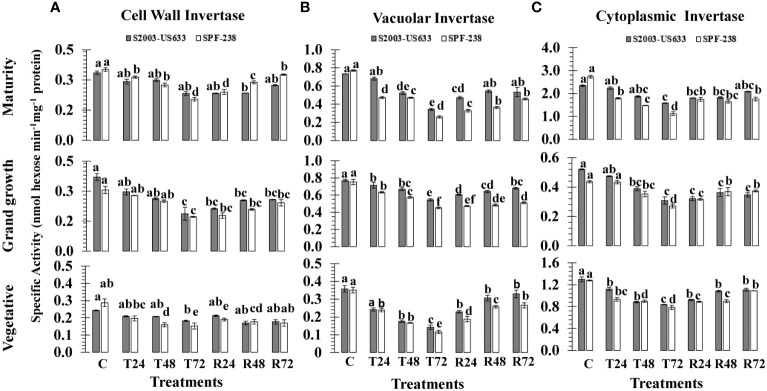
The activity of the invertase isozymes **(A)** cell wall, **(B)** vacuolar and **(C)** cell wall was measured in sugarcane cultivars S2003-US-633 and SPF-238 under the following conditions: Control (C) at 30 ± 2°C, heat shock at 45 ± 2°C (T24, T48 and T72) and recovery at 30 ± 2°C (R24, R48 and R72) for 24, 48 and 72 hours at various phenologies The values are the means and standard errors of three replicates. For each growth stage, a different letter above the bars denotes a significant difference with *p< 0.05*.

Heat stress changed the expression of invertase isozymes (CWIN, CyIN and VIN) in sugarcane cultivars. When the sugarcane plant was exposed to high temperature shock at 45°C (± 2°C) for 24, 48 and 72 hours, there was a substantial decrease in invertase isozymes. Thermotolerant cultivars had a minimally declined percentage of cell wall invertase activity, as shown in thermotolerant S2003-US-633 (21%), while susceptible SPF-238 (46%), at the maturity stage. A similar trend was exhibited in the vegetative growth stage. The average decline over the control was 31.34% and 41.59% for CWIN at the vegetative stage, respectively, due to heat shock treatments. Among growth stages, CWIN activity was at its minimum at mature stages in both cultivars (**Figure 4A**).

#### Vacuolar invertase (VIN) activity

A substantial decrease in vacuolar invertase (VIN) activity was shown in sugarcane from the vegetative to the maturity stage subjected to heat stress. Comparatively, maximum VIN activity was observed at the maturity stage in SPF-238. The thermotolerant S2003-US-633 was able to sustain a relatively high VIN under heat stress conditions. When exposed to heat stress, the susceptible cultivar (SPF-238) found the maximum fold reduction in VIN activity over the control and recovery conditions at all stages. VIN was found to be very sensitive to other isozymes at the vegetative stage (**Figure 4B**).

#### Cytoplasmic invertase (CyIN) activity

Among the sucrose metabolizing enzymes, the cytoplasmic invertase (CyIN) enzyme is one of them, which is involved in sucrose catabolism and plays a significant role in glucose and fructose synthesis in sugarcane. At T72, the mean CyIN activity was 0.839 nmol hexose min^-1^ mg^-1^ protein, which was lower in the thermotolerant cultivar (S2003-US-633) than in the susceptible cultivar (SPF-238) at the vegetative stage. A similar trend was observed with rapidly declining enzymatic activity under heat stress treatments at the grand growth stage in both cultivars. The variability in terms of CyIN activity existed in both cultivars, with the highest in control and recovery conditions at the maturity stage, while the lowest CyIN activity was noted under heat stress at the grand growth stage. Among the growth stage studies, CyIN activities were less at the grand growth stage in SPf-283 while higher in variety S2003-US-633 at the ripening stage (**Figure 4C**).

### Qualitative analysis of invertase isozymes

#### Cell wall invertase expression

**Figure 5:**
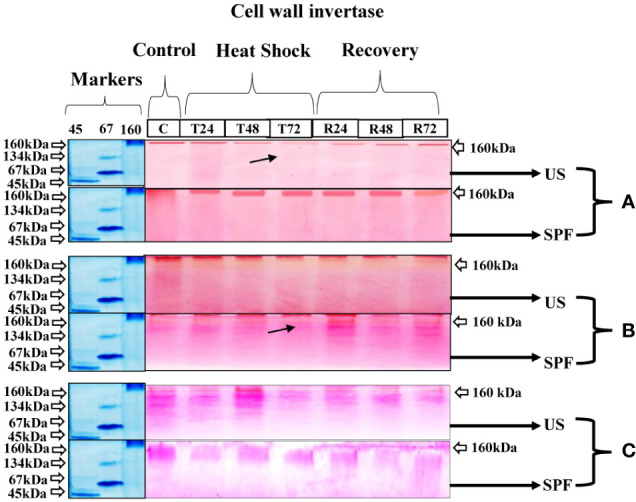
Differential expression analysis of cell wall invertase of both cultivars S2003-US-633 (US) and SPF-238 (SPF) was subjected to control (C) at 30 ± 2°C, heat shock at 45 ± 2°C (T24, T48 and T72) and recovery at 30 ± 2°C (R24, R48 and R72) for 24, 48 and 72 hours at **(A)** vegetative, **(B)** grand growth and **(C)** mantrity stages. (Markers used ovalbumin (45 kDa), albumin bovine (monomer 67 kDa and dimer 134 kDa) and gama globulin human (160 kDa). Black arrows indicate the low expression of enzyme activity during heat shock.

At all growth stages, 160 kDa of cell wall invertase CWINV was discovered. The initial expression under the heat stress condition was identical to that under the control condition at T24 (24 hours); however, following T2 (48 hours) and T3 (72 hours), the cell wall invertase band intensity declined. For cultivar S2003-US-633 under recovery conditions, a quick recovery was seen. At the vegetative stage, this cultivar, SPF-238, performed better under stressful circumstances. However, at the grand growth maturity stage (**Figure 5**), the heat stress had only a minor impact. Both cultivars that are involved in thermotolerance showed only one type of molecular-weight protein band (160 kDa) at various developmental stages.

#### Cytoplasmic invertase expression

**Figure 6:**
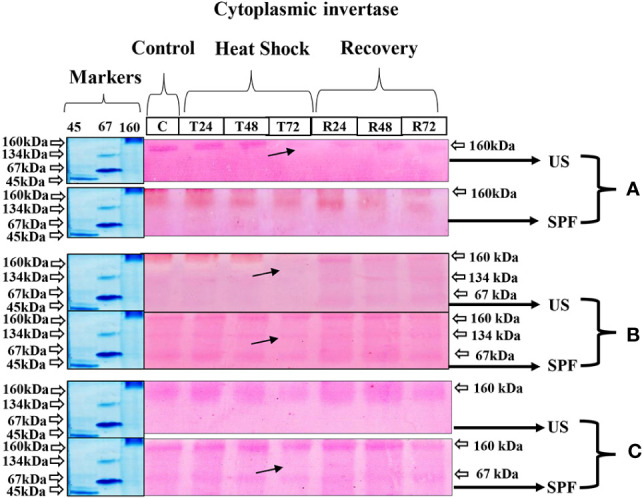
Differential expression analysis of cyntoplasmic invertase of both cultivars S2003-US-633 (US) and SPF-238 (SPF) was subjected to control (C) at 30 ± 2°C, heat shock at 45 ± 2°C (T24, T48 and T72) and recovery at 30 ± 2°C (R24, R48 and R72) for 24, 48 and 72 hours at **(A)** vegetative, **(B)** grand growth and **(C)** mantrity stages. (Markers used ovalbumin (45 kDa), albumin bovine (monomer 67 kDa and dimer 134 kDa) and gama globulin human (160 kDa). Black arrows indicate the low expression of enzyme activity during heat shock.

The zymography analysis of neutral invertase enzymes of both varieties expressed a molecular mass of 160 kDa at all growth stages. When heat stress was applied, the expression of this enzyme gradually declined over 72 hours as compared to control and recovery conditions. The same molecular weight of 160 kDa was observed in SPF-238 at all growth stages. However, at the grand growth stage, different molecular weights of protein (67,134 and 160 kDa) were observed in both varieties. Extreme temperatures severely affected cytoplasmic invertase expression at 72 hours. At recovery conditions, band intensity indicated that the CyIN expression level was higher at the grand growth stage than at other growth stages (**Figure 6**).

#### Vacuolar invertase expression

**Figure 7:**
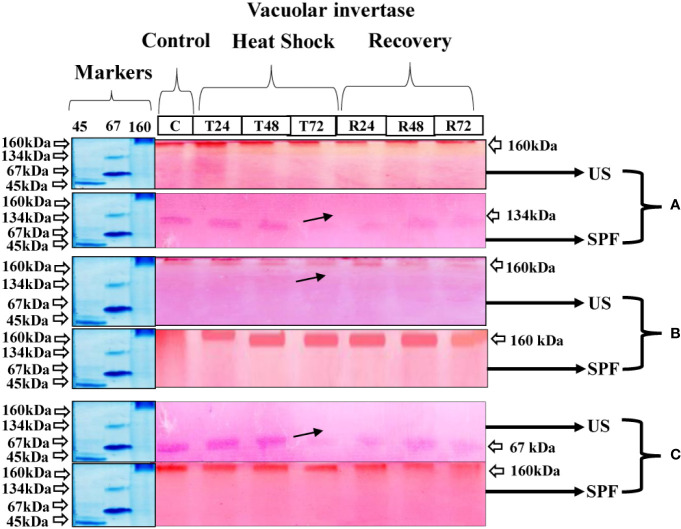
Differential expression analysis of vacoular invertase of both cultivars S2003-US-633 (US) and SPF-238 (SPF) was subjected to control (C) at 30 ± 2°C, heat shock at 45 ± 2°C (T24, T48 and T72) and recovery at 30 ± 2°C (R24, R48 and R72) for 24, 48 and 72 hours at **(A)** vegetative, **(B)** grand growth and **(C)** mantrity stages. (Markers used ovalbumin (45 kDa), albumin bovine (monomer 67 kDa and dimer 134 kDa) and gama globulin human (160 kDa). Black arrows indicate the low expression of enzyme activity during heat shock.

At the vegetative stage, cultivar S2003-US-633 showed little variation in the pattern of vacuolar invertase expression in response to heat stress. The molecular weight of this cultivar was 160 kDa for vegetative and grand growth and 67 kDa at maturity. However, the VIN expression pattern was better at 160 kDa at the grand growth stage compared to other growth stages. However, the expression of these enzymes is significantly hampered at the maturity stage following a 72-hour heat shock treatment. In cultivar SPF-238, 134 kDa, maximal band intensity was seen in the untreated crop. However, with heat stress, the VIN activity steadily decreased and became seriously damaged after 72 hours, when the crop was in the vegetative stage. Although the 160 kDa molecular mass was visible in both the control and treatment groups, VIN was expressed less at the maturity stage than at the grand growth stage (**Figure 7**).

### Sugar parameter estimation

#### The sugar recovery rate

Sugar recovery rates declined under thermal stress. Data herein presented in **Table 1** shows that in all studies of sugarcane cultivars, the sugar recovery rate varied significantly between 14.49% in S2003-US-633 and 13.68% in SPF-238 in untreated plants, whereas under stress, the sugar recovery rate declined by a percentage ranging from 13.64–12.93% (S2003–US-633) and 13.20–12.18% (SPF–238) at maturity stage. Similar trends were noted in pol and brix concentrations. On the contrary, the maximum fiber content was found in SPF-238.

**Table 1:** Quality parameters of both cultivars S2003-US-633 and SPF-238 under control (30±2°C), heat shock (45±2°C) and recovery (30±2°C) for 2 24, 48 and 72h at grand growth and maturity stages.

Grand Growth Stage													
Parameters	Varieties	Mean ± SEM									*P Value*		
		Control		Heat shock					Recovery		Cultivar	Treatment	Interaction
		C	T24	T48			T72	R24	R48	R72	C	T	C×T
**°Brix**	S2003-US-633 SPF-238	13.78±0.12 13.44±0.19	12.85±0.37 13.12±0.06	12.82±0.06 12.25±0.10			12.41±0.13 11.88±0.07	12.14±0.11 12.44±0.15	12.52±0.13 12.46±0.12	12.74±0.05 12.56±0.12	*p<0.05*	*p<0.05*	*p<0.05*
**Fiber content (% )**	S2003-US-633 SPF-238	17.55±0.40 18.89±0.35	15.48±0.70 17.22±0.29	14.51±0.38 17.08±0.58			13.92±0.29 16.45±0.39	15.19±0.39 14.56±0.15	16.81±0.33 17.87±0.98	16.92±0.33 18.44±0.60	*p<0.05*	*p<0.05*	*p<0.05*
**Polarization**	S2003-US-633 SPF-238	12.13±0.66 10.25±0.19	10.76±0.25 10.05±0.27	10.31±0.24 9.31±0.130			9.47±0.245 9.00±0.329	9.36±0.250 9.36±0.004	10.28±0.18 9.39±0.011	9.81±0.325 10.35±0.01	*p<0.05*	*p<0.05*	*p<0.05*
**Recovery (% )**	S2003-US-633 SPF-238	9.63±0.66 7.75±0.18	8.26±0.253 7.55±0.272	7.81±0.245 6.81±0.130			6.97±0.246 6.50±0.329	6.86±0.251 6.86±0.001	7.78±0.187 6.89±0.015	7.31±0.325 7.58±0.007	*p<0.05*	*p<0.05*	*p<0.05*
Maturity													
**°Brix**	S2003-US-633 SPF-238	18.13±0.48 16.69±0.48	17.41±0.28 16.41±0.67	17.40±0.35 16.16±0.59			16.65±0.62 15.48±0.71	16.40±0.65 15.54±0.33	17.20±0.66 16.18±1.03	17.64±0.58 16.14±1.03	*p<0.05*	*p>0.05*	*p>0.05*
**Fiber content (% )**	S2003-US-633 SPF-238	14.40±1.71 18.68±0.73	13.39±0.28 17.71±0.67	11.60±0.36 15.17±0.31			11.35±0.62 14.52±0.13	11.60±0.66 14.79±0.58	11.39±0.67 15.82±1.00	11.86±0.59 15.59±1.03	*p<0.05*	*p<0.05*	*p>0.05*
**Polarization**	S2003-US-633 SPF-238	16.99±0.25 16.18±0.08	16.14±0.06 15.70±0.18	15.60±0.06 14.83±0.15			15.43±0.20 14.68±0.08	15.53±0.36 15.18±0.30	15.99±0.50 15.70±0.76	16.08±0.40 16.00±0.88	*p<0.05*	*p<0.05*	*p>0.05*
**Recovery (% )**	S2003-US-633 SPF-238	14.49±0.25 13.68±0.08	13.64±0.06 13.20±0.18	13.10±0.87 12.33±0.15			12.93±0.25 12.18±0.08	13.03±0.36 12.37±0.24	13.49±0.49 31.21±0.75	13.58±0.39 13.50±0.87	*p<0.05*	*p<0.05*	*p>0.05*

C, Cultivar; T, Treatments; C×T, Cultivar×Treatments; at *p level p<0.05*.

#### Sugars analysis

Both sugarcane cultivars showed a decrease in total sugar content when subjected to thermal stress, with the highest and lowest folding percentage increases occurring in S2003-US-633 (10818.3 mg g^-1^ FW) and SFP-238 (8573.3 mg g^-1^ FW). There is a substantial decline (8633.1 mg g^-1^ FW) and (7027.6 mg g^-1^ FW) when exposed to heat stress for 72 hours, respectively. The same trend was noted in reducing sugars in both cultivars. Comparatively, maximum reducing sugar was found in SFP-238, while maximum total sugar was noted in S2003-US-633 at the maturity stage. Regarding non-reducing sugar, same trends as total sugars content was observed in both cultivars at all stages see **Table 2**.

**Table 2:** Sugar analysis of both cultivars S2003-US-633 and SPF-238 under control (30 ± 2°C), heat shock (45 ± 2°C) and recovery (30 ± 2°C) for 24, 48 and 72h at vegetative, grand growth and maturity stages. at *p level p<0.05*.

Vegetative stage											
Parameters	Varieties	Mean ± SEM							*P Value*		
		Control	Heat shock			Recovery			Cultivar	Treatment	Interaction
		C	T24	T48	T72	R24	R48	R72	C	T	C×T
**Total sugars (mg g^-1^ FW)**	S2003-US-633	712.64 ± 15.2	391.81 ± 15.4	345.0 ± 5.85	263.15 ± 44.2	309.8 ± 15.4	584.7 ± 42.5	608.1 ± 45.7	*p<0.05*	*p<0.05*	*p>0.05*
	SPF-238	573.09 ± 25.5	333.33 ± 36.5	286.55 ± 32.5	228.07 ± 10.1	304.09 ± 46.8	415.20 ± 25.5	578.94 ± 20.2			
**Reducing sugar** **(mg g^-1^ FW)**	S2003-US-633	0.25 ± .008	0.15 ± .08	0.13 ± .00	0.12 ± .00	0.19 ± .01	0.20 ± .01	0.21 ± .01	*p<0.05*	*p<0.05*	*p>0.05*
	SPF-238	0.19 ± .016	0.15 ± .06	0.12 ± .00	0.11 ± .00	0.18 ± .00	0.18 ± .01	0.18 ± .02			
**Non-Reducing sugar** **(mg g^-1^ FW)**	S2003-US-633	0.45 ± .017	0.24 ± .018	0.2 ± .002	0.14 ± .046	0.1 ± .009	0.37 ± .041	0.39 ± .06	*p<0.05*	*p<0.05*	*p>0.05*
	SPF-238	0.37 ± .037	0.18 ± .039	0.16 ± .034	0.11 ± .01	0.12 ± .047	0.233 ± .027	0.39 ± .03			
Grand growth Stage											
**Total sugars (mg g^-1^ FW)**	S2003-US-633	1641.0 ± 39.21	1623.9 ± 30.8	1367.5 ± 22.6	1213.6 ± 30.8	1230.7 ± 39.2	1316.2 ± 47.6	1461.5 ± 25.6	*p<0.05*	*p<0.05*	*p>0.05*
	SPF-238	1581.1 ± 42.7	1402.2 ± 23.0	1241.3 ± 39.8	1195.4 ± 23.0	1213.6 ± 30.8	1307.6 ± 14.8	1410.2 ± 25.6			
**Reducing sugar** **(mg g^-1^ FW)**	S2003-US-633	0.52 ± .02	0.48 ± .00	0.38 ± .01	0.31 ± .01	0.34 ± .01	0.40 ± .00	0.45 ± .02	*p<0.05*	*p<0.05*	*p>0.05*
	SPF-238	0.61 ± .01	0.58 ± .01	0.58 ± .01	0.44 ± .03	0.45 ± .03	0.52 ± .02	0.57 ± .01			
**Non-Reducing sugar** **(mg g^-1^ FW)**	S2003-US-633	0.4 ± .017	0.2 ± .018	0.21 ± .002	0.14 ± .046	0.11 ± .009	0.37 ± .041	0.39 ± .066	*p<0.05*	*p<0.05*	*p>0.05*
	SPF-238	0.18 ± .012	0.12 ± .018	0.15 ± .034	0.11 ± .010	0.123 ± .047	0.23 ± .027	0.39 ± .036			
Maturity Stage											
**Total sugars (mg g^-1^ FW)**	S2003-US-633	10818.3 ± 70	9471.8 ± 326	9044.4 ± 178	8663.1 ± 371	9020.3 ± 426	9298.7 ± 108	9714.2 ± 58	*p<0.05*	*p<0.05*	*p>0.05*
	SPF-238	8573.3 ± 340	7020.9 ± 874	6955.9 ± 178	7027.6 ± 205	7392.9 ± 128	7131.7 ± 177	7388.5 ± 46			
**Reducing sugar** **(mg g^-1^ FW)**	S2003-US-633	0.23 ± .001	0.23 ± .007	0.24 ± .017	0.23 ± .006	0.23 ± .003	0.22 ± .004	0.23 ± .022	*p<0.05*	*p>0.05*	*p>0.05*
	SPF-238	0.35 ± .001	0.30 ± .013	0.36 ± .045	0.32 ± .012	0.31 ± .003	0.36 ± .024	0.33 ± .007			
**Non-Reducing sugar** **(mg g^-1^ FW)**	S2003-US-633	10.6 ± .07	9.2 ± .32	8.8 ± .38	8.4 ± .37	8.8 ± .43	9.1 ± .11	9.5 ± .08	*p<0.05*	*p<0.05*	*p>0.05*
	SPF-238	8.2 ± .35	6.7 ± .89	6.6 ± .38	6.7 ± 19	7.1 ± .13	6.8 ± .18	7.1 ± .08			

#### Morphological analysis

The biomass of both cultivars decreased with exposure to heat stress. Specifically, cultivar SPF-238 had a lower fresh weight to dry weight ratio than S2003-US-633. Cultivar S2003-US-633 had longer shoot and root lengths than SPF-238. This result indicates that the S2003-US-633 cultivar had better performance in heat stress conditions at all phenologies (see **Table 3**).

**Table 3:** Morphological parameters of both cultivars S2003-US-633 and SPF-238 under control at (30 ± 2°C), heat shock (45 ± 2°C) and recovery (30 ± 2°C) for 24, 48 and 48 h at various phenology.

Vegetative Stage											
Parameters	Varieties	Mean ± SEM							*P Value*		
		Control	Heat shock			Recovery			Cultivar	Treatment	Interaction
		C	T24	T48	T72	R24	R48	R72	C	T	C×T
**Shoot length (cm)**	S2003-US-633	89.00 ± 0.56	88.55 ± 0.22	88.44 ± 0.77	87.22 ± 0.77	87.00 ± 0.65	86.55 ± 0.32	87.55 ± 0.32	*p<0.05*	*p<0.05*	*p<0.05*
	SPF-238	87.00 ± 0.77	86.00 ± 0.47	83.00 ± 0.56	83.00 ± 0.55	83.00 ± 0.55	83.22 ± 0.22	83.00 ± 0.55			
**Root length (cm)**	S2003-US-633	18.0 ± 0.20	18.2 ± 0.20	18.1 ± 0.60	17.0 ± 0.80	17.0 ± 0.20	18.0 ± 0.00	18.0 ± 0.00	*p<0.05*	*p<0.05*	*p>0.05*
	SPF-238	17.2 ± 0.20	17.1 ± 0.20	16.0 ± 0.00	15.0 ± 0.00	16.0 ± 0.00	16.2 ± 0.10	16.1 ± 0.20			
**Fresh to dry wt ratio (%)**	S2003-US-633	26.93 ± 0.30	26.00 ± 0.01	25.01 ± 0.73	23.99 ± 0.55	24.33 ± 0.33	24.33 ± 0.77	24.00 ± 0.00	*p>0.05*	*p<0.05*	*p>0.05*
	SPF-238	25.48 ± 0.58	24.12 ± 0.33	24.01 ± 1.33	23.77 ± 0.22	24.44 ± 0.22	24.96 ± 0.50	25.03 ± 0.42			
Grand Growth Stage											
**Shoot length (cm)**	S2003-US-633	166.2 ± 0.22	166.6 ± 0.30	165.3 ± 0.44	165.1 ± 0.00	165.1 ± 0.11	165.2 ± 0.22	165.9 ± 0.99	*p<0.05*	*p>0.05*	*p>0.05*
	SPF-238	164.5 ± 0.50	165.2 ± 1.00	164.30 ± 0.30	164.31 ± 0.32	164.5 ± 1.44	165.0 ± 0.77	165.50 ± 0.77			
**Root length (cm)**	S2003-US-633	40.0 ± 0.48	35.50 ± 2.0	34.66 ± 2.0	34.00 ± 0.00	34.0 ± 1.0	34.00 ± 0.66	34.33 ± 0.67	*p<0.05*	*p<0.05*	*p>0.05*
	SPF-238	37.10 ± 1.00	37.0 ± 0.66	35.30 ± 0.30	35.00 ± 0.66	35.88 ± 0.90	36.0 ± 0.00	36.30 ± 0.30			
**Fresh to dry wt ratio (%)**	S2003-US-633	38.55 ± 1.0	37.0 ± 0.66	35.6 ± 1.20	37.7 ± 0.66	38.0 ± 0.00	38.0 ± 2.00	38.2 ± 0.2.00	*p>0.05*	*p<0.05*	*p<0.05*
	SPF-238	43.0 ± 2.0	41.33 ± 1.33	38.7 ± 1.00	37.0 ± 0.00	37.6 ± 2.33	36.0 ± 2.00	36.5 ± 3.33			
Maturity Stage											
**Shoot length (cm)**	S2003-US-633	316.33 ± 2.0	315.3 ± 0.33	315.2 ± 0.77	314 ± 2.00	314 ± 0.00	314.6 ± 0.66	315.44 ± 0.77	*p>0.05*	*p<0.05*	*p>0.05*
	SPF-238	315.22 ± 0.22	314.11 ± 0.11	313.44 ± 0.32	312.7 ± 0.77	313.7 ± 0.44	314.2 ± 0.22	315.0 ± 0.00			
**Root length (cm)**	S2003-US-633	81.00 ± 2.00	80.00 ± 0.50	77.00 ± 1.0	75.00 ± 1.0	76.00 ± 1.0	77.00 ± 1.0	78.00 ± 2.0	*p<0.05*	*p<0.05*	*p<0.05*
	SPF-238	77.00 ± 0.40	76.00 ± 1.0	76.00 ± 1.50	75.00 ± 0.20	75.00 ± .1.0	76.00 ± 0.60	76.00 ± 0.50			
**Fresh to dry wt ratio (%)**	S2003-US-633	43.0± 2.20	39.88 ± 0.38	38.30 ± 2.00	36.90 ± 0.00	37.15 ± 0.34	37.75 ± 0.60	39.00 ± 0.50	*p<0.05*	*p<0.05*	*p>0.05*
	SPF-238	39.44 ± 0.44	38.88 ± 0.55	37.55 ± 0.50	36.05 ± 0.44	36.55 ± 0.40	36.89 ± 1.55	37.23 ± 0.22			

C, Cultivar; T, Treatments; C×T, Cultivar×Treatments; at *p level p<0.05*.

#### Correlation analysis

**Figure 8:**
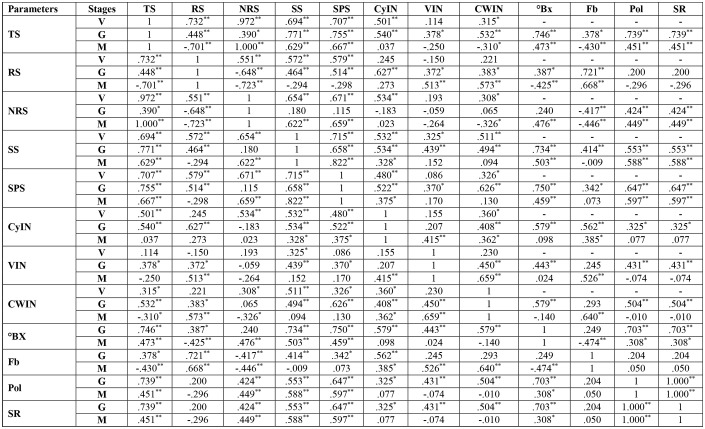
Correlation analysis between sucrose metabolizing enzymes and sugar quality parameters of two cultivars, S2003-US-633 and SPF-238, at vegetative, grand growth and maturity stages. SPS, Sucrose Phosphate synthase; SS, Sucrose synthase; CWIN, Cell Wall Invertase; CyIN, Cytoplasmic Invertase; VIN, Vacuolar Invertase; RS, Reducing Sugar; Pol, Polarization; Fb, Fiber Content; ⁰Bx, ⁰Brix; SR, Sugar Recovery; TS, Total Sugar; V, Vegeative; G, Grand Growth; M, Manturity. **Significant correlation at p<0.01 and *Significant correlation at p<0.05.

At the vegetative stage, in cultivar S2003-US-633, SS, SPS and TS had a strongly negative correlation with VIN, CyIN and RS. But the association between total sugar and SS and SPS was a strongly positive correlation. In cultivar, SPF-238, SS and TS were strongly correlated with RS, CWIN and CyIN. Only SPS was weakly correlated with reducing sugar, CWIN and CyIN. At the grand growth stage, invertase isozymes were strongly negatively associated with SPS, SS and total sugar in both varieties. At the ripening or maturity stage, total sugar (TS) was strongly positively correlated with SS and SPS, while there was a strongly negative correlation with invertase isozymes, fiber content (Fb) and reducing sugar (RS) in both cultivars (**Figure 8**).

#### Heat map

**Figure 9:**
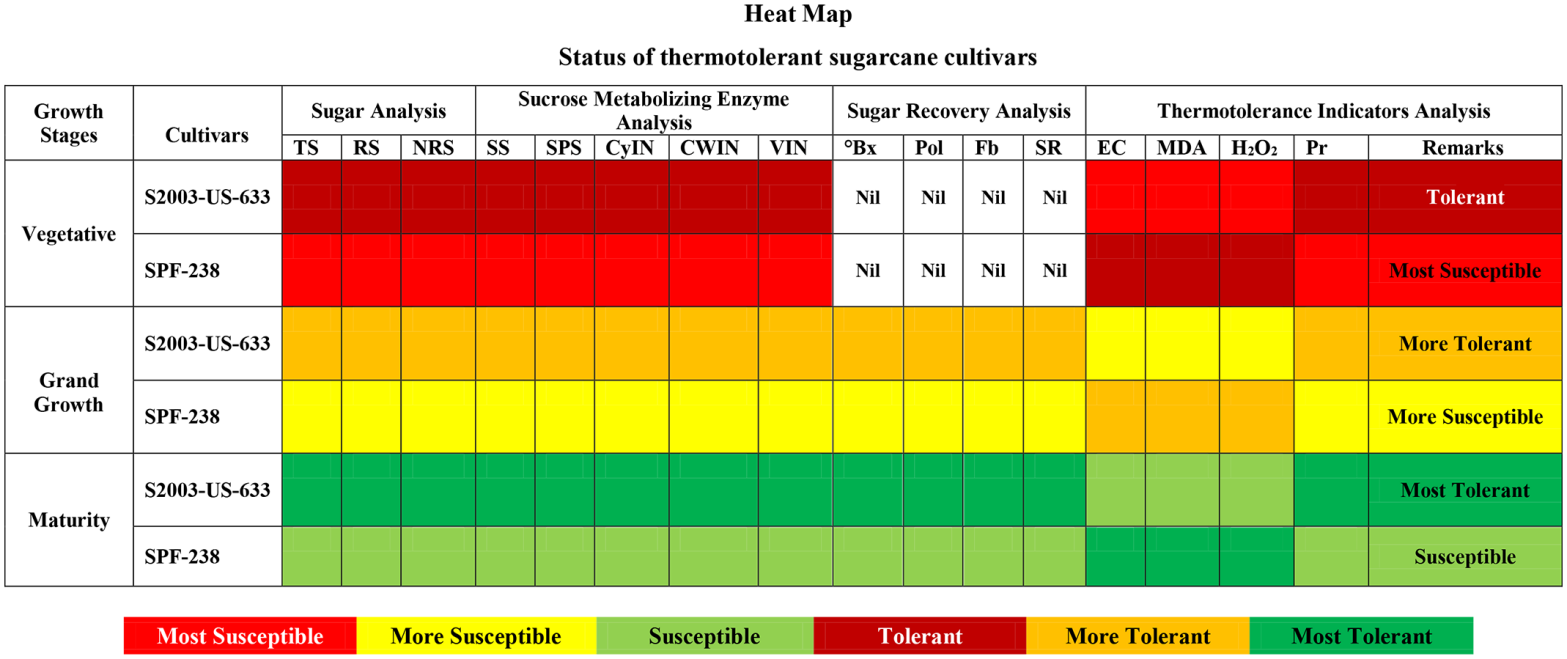
Heat-map represents the thermotolerant status of both cultivars of sugarcane at different phenological phases. TS, Total Sugar; RS, Reducing Sugar; NRS, Non-reducing Sugar; SS, Sucrose Synthase; SPS, Sucrose Phosphate Synthase; CyIN, Cytoplasmic Invertase; CWIN, Cell Wall Invertase; VIN;, Vacuolar Invertase; °Bx, °Brix; SR, Sugar Recovery; Pol, Polarization; Fb, Fiber; Pr, Proline.

The heat map represents the thermotolerant cultivars of sugarcane. The dark red color indicates tolerant, the dark yellow indicates more tolerant, and the dark green color indicates the most tolerant cultivar (S2003-US-633). While the light green color indicates susceptible, the light yellow color is more susceptible, and the light red color is the most susceptible cultivar (SPF-238) at all growth stages (**Figure 9**).

#### Schematic model of sucrose metabolizing enzyme expression and sugar content

**Figure 10:**
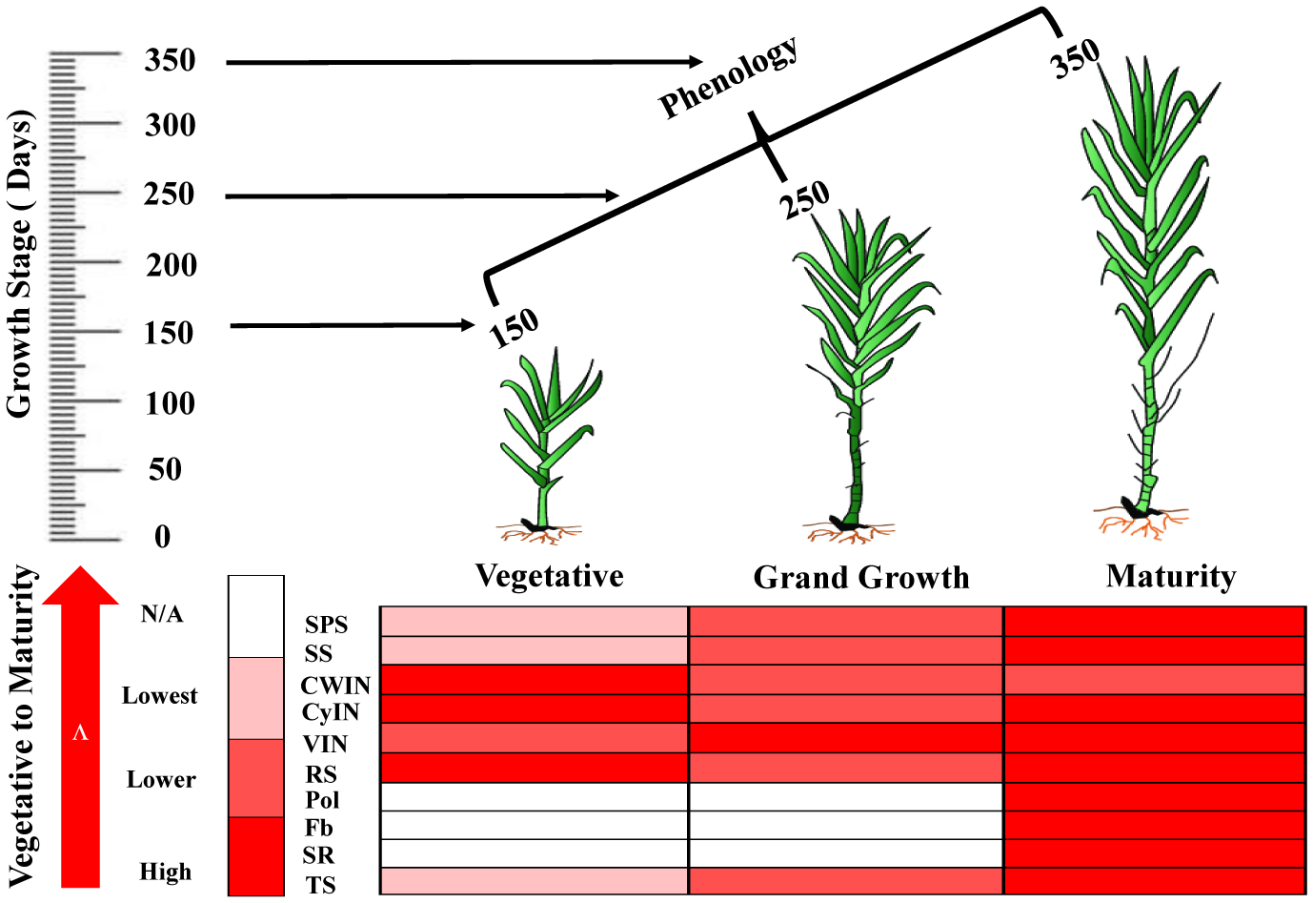
The biological map represents the expression pattern of sucrose metabolizing enzymes and sugar’s quality parameters in sugarcane at different growth stages. SPS, Sucrose Phosphate Synthase; SS, Sucrose Synthase; CWIN, Cell Wall Invertase; CyIN, Cytoplasmic Invertase; VIN, Vacuolar Invertase; RS, Reducing Sugar; Pol, Polarization; Fb, Fiber Content; SR, Sugar Recovery; TS, Total Sugar.

The schematic model represents the expression of sucrose metabolizing enzymes such as SPS, SS, invertase isozymes (CWIN, CyIN and VIN) and sugar content at different phenologies of sugarcane (**Figure 10**).

## Discussion

In sugarcane, sucrose is transported from the leaf to accumulation in the stem via different pathways along with transporter proteins and sugar-metabolizing enzymes. These enzymes play a vital role during sucrose synthesis and storage. A common trend among invertase isozymes in the course of sugarcane development is that these isozymes tend to be highly expressed in juvenile leaves, with expression decreasing significantly at the maturity stage. However, SPS and SS were highly expressed at the maturity stage, regardless of whether the enzymes were functioning in sucrose synthesis or hydrolysis. Previous results were also reported in corn, sugarcane (Claussen et al., 1985) and sugar beet (Pavlinova et al., 2002). The synchronized alteration of sucrose synthesis and hydrolysis activity helps maintain sucrose levels within a suitable concentration in sugarcane leaves and stems, a crucial function critical for usual plant growth and development (Yadav et al., 2014).

In plants, SPS activity is normally involved in sucrose synthesis (Anur et al., 2020), while SS activity is mostly involved in sucrose cleavage or resynthesis (Winter and Huber, 2000) and it also helps in the equilibrium of sugar in plant cells (Stein and Granot, 2019). From the vegetative to the mature stages, SS activity increased. The same results were also reported (Tana et al., 2014). Contrarily, sucrose synthase activities and their gene expression are substantially higher in young sugarcane internodes, possibly providing carbon for cellulose and cell walls synthesized from uridine-diphosphate-glucose (UDP-G) (Mason et al., 2023). A substantial decrease in SPS-specific activity was exhibited in both varieties, suggesting adverse effects on enzyme functions in sugarcane cultivars due to heat shock treatments, which were also reported in another genotype of sugarcane (Neliana et al., 2019).This finding suggested that sugarcane plants demand optimum temperatures for survival, especially for maximum sucrose accumulation in the stem at the maturity stage. Maximum SPS expression was exhibited at the maturity stage and the correlation analysis showed that the sucrose content had a strongly positive association with SPS. In contrast, despite SPS mRNA levels being substantially higher in mature leaves than immature ones, SPS activity remained remarkably stable throughout the developmental phases (Suzue et al., 2006). Post-transcriptional changes of the enzymes may be crucial in regulating enzyme activity (SPS) (Walker and Huber, 1989). Regarding heat shock, it severely affected the SS and SPS activity at all growth stages. Earlier studies have also stated that temperature is a key factor for maximum enzymatic activity; SPS and SS are both optimized at 37°C (Verma et al., 2019). Recently, another study on sugarcane explained that the heat stress severely affected the SPS and SS, which decreased the sucrose (Shanthi et al., 2023).

In the case of invertase isozymes, neutral invertase expression was comparable to the other two invertase isozymes (VIN and CWIN) throughout the developmental stages, suggesting an active contribution of invertase isozymes in sugarcane development. Of the five sucrose-metabolizing enzymes, only invertase isozymes (NIV, CWIN and VIN) observed a negative association at a significant level for their expression with sucrose content at grand growth and maturation stages. Further supporting its role in this process, 8 out of 10 neutral invertase genes were reported in Hevea (Liu et al., 2015), providing further evidence for the dynamic role of NIV in development in the sugarcane plant. Qualitative analysis (Zymography) revealed different molecular weight proteins, such as 67 kDa, 134 kDa and 160 kDa, were expressed at different growth stages in both cultivars. These findings suggest that the invertase isozymes have different roles at different locations in plant cells, along with different growth stages.

Previous studies described that under ecological stress, the coordinated action of sucrose-metabolizing enzymes largely determines the capacity of crops to synthesize sucrose and the concentration of sucrose in leaves (Rosales et al., 2007). Sucrose has been suggested to become a reactive oxygen species scavenger in Arabidopsis at high concentrations, contrary to its minimal concentrations when it functions as a signaling molecule (Sugio et al., 2009). It was also assumed that the activities of sucrose-metabolizing enzymes under thermal stress decreased with sucrose concentration (Ebrahim et al., 1998). According to our data, at maturity, vacuolar invertase activity was declining in S2003-US-633, suggesting that this cultivar has strong sink strength. This study’s findings were in agreement with earlier research on sugar beet, which found that juvenile leaves had higher levels of vacuolar invertase activity than adult leaves (Pavlinova et al., 2002). Contrary to what one might expect, more invertase activity is associated with higher sucrose content in sugarcane (Wang et al., 2023). The sucrose resynthesis and hydrolysis model as proposed by Glasziou and Glayer may explain this (Glasziou and Gayler, 1972). But the high expression of the CWIN gene suggests that there is no proof that significant amounts of sugar are being synthesized again. It was further reported that the maximum expression of the cell wall (CWI4) and vacuolar invertase (ANINV1-1 and 3) gene families suggests that sucrose cleavage is a significant competitive factor for sink strength (Chen et al., 2015; Mason et al., 2023). Recently, five CWIN genes (CWIN1–5) were reported in grape berries (Du et al., 2023). On the other hand, invertase cleaves sucrose into hexose for growth and development in young tissues at the formative and growth stages (Chandra et al., 2012). Vacuolar invertase expression is controlled by sugars (Sturm and Tang, 1999) as well as a variety of biotic and abiotic stress factors (Godt and Roitsch, 1997). A recent study found that the Chinese herb *Dendrobium officinale* contains four genes for acid invertase (DoAINV) that are involved in plant growth, cell elongation, various stress responses and polysaccharide production (Liu et al., 2023). Additionally, the study claimed that invertase had a role in the metabolism of sucrose and the source-sink relationship in the tree peony’s leaves and buds (Wang et al., 2023). Our study showed that maximum vacuolar invertase activity was exhibited in SPF-238 as compared to S2003-US-633 at the maturity or ripening stage, which indicates that it is a low sink strength cultivar because the sucrose content declines due to the higher invertase activity, which enhances the reducing sugar in the sugarcane stem. This decline may be due to increased reactive oxygen species (ROS) in plant cells under heat stress because when ROS increase, the enzymatic or metabolic activity disfunctions, which leads to a decline in sugars (Liu et al., 2016). However, in the case of S2003-US-633, rather than SPF-238, S2003-US-633 had the lowest level of VIN activity. Additionally, correlation analysis (**Figure 8**) supported the notion that invertase activities were inversely correlated with SPS and sucrose content. According to this finding, the significant sucrose accumulation in S2003-US-633 was caused by its low rate of hydrolysis into hexose, thermotolerance and high sink strength.

The average lipid peroxidation enhancement over the controlled condition was threefold greater in both cultivars due to heat shock treatments. The thermotolerant index of lipid peroxidation was higher in the thermotolerant cultivar S2003-US-633 than in another cultivar. Prior research has demonstrated that the production of antioxidant enzymes in sugarcane is related to cultivars’ relative tolerance to heat stress, as seen by their lower levels of lipid peroxidation and membrane stability (Abbas et al., 2013). In the study on the opium poppy plant, the MDA content was inversely proportional to antioxidants (Zhao et al., 2010). Lipid peroxidation significantly increases under conditions of high temperature stress (Gomathi et al., 2013).These findings suggest that under heat stress conditions, lipid peroxidation leads to reactive oxygen species production in plant cells along with membrane integrity loss (Blokhina et al., 2003; Boaretto et al., 2014).

The plasma membrane is the primary site for injury in plant cells, which can be measured by ion leakage (Blum, 2018). Plant cells have an optimum temperature for growth and development and exceeding this temperature can lead to cell death. A recent study observed damage to the thermostability of the plant cell membrane, or plasma membrane, of susceptible cultivar SPF-238, while tolerant cultivar S2003-US-633 maintained thermostability with minimal electrolyte leakage. Membrane integrity is lost, unsaturated fatty acids and Ca^2+^ influx are increased and electrolyte leakage is connected with crop yield reduction (ElBasyoni et al., 2017; Khan et al., 2019). Pathogen attack, salinity and heat stress all cause cell membrane thermostability, which is dependent on species and cell types (Demidchik et al., 2010). High temperatures caused the overproduction of ROS, which affected photosynthetic machinery and physiological and biochemical functions, leading to a decline in crop yield and quality (Sharma et al., 2017; Li et al., 2018). Hydrogen peroxide is essential for photosynthesis, cell wall cross-linkage, stress acclimation and the oxidative defense system (Rodrigues et al., 2017). Hydrogen peroxide content was highest in both varieties under high temperature stress, while it was minimal in S2003-US-633, indicating high integrity of the cell membrane. Reactive oxygen species, including hydrogen peroxide, typically cause harm to vital biological components when their levels rise (Mittler, 2006). Under high temperatures, similar outcomes were also seen in wheat and canola plants (Akram et al., 2018). Earlier studies reported a similar result in wheat (Kumar et al., 2012) and sugarcane plants (Kohila and Gomathi, 2018).

In cultivar S2003-US-633, maximum proline accumulations of 70%, 60% and 67% were noted under thermal stress at the vegetative, grand growth and maturity stages, respectively. This finding suggested that the maximum accumulation of proline under heat shock treatments could act as chaperones and reactive oxygen scavengers, protecting cellular mechanisms such as enzymes, maintaining water levels in cells and membranes and providing carbon for plants under stress conditions (Hameed et al., 2012). During high-temperature stress conditions, proline supplies energy for respiration and ammonia sources. After the stress is relieved, proline directly contributes to plant metabolism. By lowering reactive oxygen species levels and defending cell membranes, it is crucial for reducing heat stress. In order to control osmatic activities and safeguard cellular structures, proline and other suitable solutes are crucial (Hayat and Khan, 2012; Khan et al., 2019) and proline accumulation maintains water balances in plant cells (Gupta et al., 2013). In different kinds of plants, the synthesis of proline differs (Hussain et al., 2019). Hence, according to the present study, S2003-US-633 was a cultivar with greater potential for accumulating free proline under heat stress at all growth stages. These biochemical traits can help molecular breeders select thermotolerant variants with higher sucrose content in sugarcane plants by indicating to what extent sugarcane plants can adapt to challenging environmental conditions.

Regarding sugar analysis, the present data showed the minimum sugar content found under heat stress conditions. On the contrary, the maximum total sugar content was observed under thermal stress conditions (Hassanein et al., 2012). Our finding proposes that the maximum sucrose content in S2003-US-633 may be due to the inhibition of invertase due to heat stress. Invertase activities, on the other hand, may cause more sucrose to be hydrolyzed into hexose sugar in SFP-238, resulting in a lower sucrose concentration. The current research revealed that the sucrose content initially decreased (at the vegetative stage), then slightly increased (at the grand growth stage) and finally stabilized (at the maturity stage). The study found a positive correlation between glucose content and invertase enzymes in sugarcane at the maturity stage. Further evidence supports our finding that sucrose synthase activity (SS) was positively associated with sucrose content in *Cucumis melo* (Burger and Schaffer, 2007). Sucrose synthase activity (SS) in cultivar S2003-SU-633 sugarcane remained high, with a slight decrease in activity at the vegetative stage. Sucrose phosphate synthase (SPS) expression showed an upward trend and peaked from control to recovery conditions at the maturity stage. Maximum SS and SPS activity, along with strongly positive total sugar, sugar recovery, pol, brix and a negative correlation with reducing sugar, invertase isozymes and fiber content, were shown in the cultivar (S2003-US-633) at all stages.

## Conclusions

Both sugarcane cultivars had substantial physiological and metabolic alterations as a result of heat stress at all development stages; however, the vegetative stage was more susceptible to these changes than the grand growth and maturity stages. Compared to SPF-238, the cultivar S2003-US-633 demonstrated thermotolerant behavior under heat shock. Cultivar S2003-US-633 had the highest proline, sugar content, or sugar recovery. The minimal concentrations of hydrogen peroxide, lipid peroxidation and electrolyte leakage were found in S2003-US-633, on the other hand, suggest that these are closely related to the maintenance of osmotic homeostasis in sugarcane plants under temperature stress. The several invertase isoforms that were discovered under heat stress and their related biochemical pathways provide a new possibility for the sugarcane molecular breeding program in relation to thermal stress. A donor cultivar for thermotolerance and high sucrose content, S2003-US-633, was discovered to be the most thermotolerant cultivar of the ones evaluated, having the highest SPS, SS and sucrose content, as shown by the Duncan test. For high-temperature agricultural production systems, significantly increased sucrose buildup or crop yields are predicted, which might enhance local economies based on the sugarcane industry and provide food for a large population.

## Data availability statement

The original contributions presented in the study are included in the article. Further inquiries can be directed the corresponding authors.

## Author contributions

FM: Conceptualization, Data curation, Formal Analysis, Investigation, Methodology, Project administration, Writing – original draft, Writing – review & editing. XL: Validation, Formal analysis, Funding acquisition. ZR: Methodology, Writing – review & editing. UJ: Methodology, Writing – review & editing. AA: Methodology, Writing – review & editing. SG: Conceptualization, Investigation, Project administration, Supervision, Writing – review & editing.
